# Plasma n-3 and n-6 fatty acids and inflammatory markers in Chinese vegetarians

**DOI:** 10.1186/1476-511X-13-151

**Published:** 2014-09-29

**Authors:** Xiaomei Yu, Tao Huang, Xiumei Weng, Tianxing Shou, Qiang Wang, Xiaoqiong Zhou, Qinxin Hu, Duo Li

**Affiliations:** Department of Clinical Laboratory, Zhejiang Hospital, 12 Linyin Road, Hangzhou, 310030 China; Department of Nutrition, Harvard School of Public Health, Boston, USA; Department of Food Science and Nutrition, Zhejiang University, 866 Yuhangtang Road, Hangzhou, 310059 China

**Keywords:** Polyunsaturated fatty acid, Vegetarians, Omnivores, Inflammatory factor

## Abstract

**Background:**

Polyunsaturated fatty acid (PUFA) intake favorably affects chronic inflammatory-related diseases such as cardiovascular disease; however, the relationship between the PUFA and inflammatory factors in the healthy vegetarians were not clear. We aimed to investigate the plasma fatty acids status, and its association with plasma inflammatory factors in Chinese vegetarians and omnivores.

**Methods:**

A total of 89 male vegetarians and 106 male omnivores were participated the study. Plasma concentrations of inflammatory factors were detected by ELISA, and as standard methods fatty acids were extracted and determined by chromatography.

**Results:**

Compared with omnivores, vegetarians have significant higher interleukin-6 (IL-6), plasma n-6 PUFA, n-6/n-3, and 18:3n-3; while they have significant lower leukotriene B4 (LTB4), cyclo-oxygenase-2 (COX2) and prostaglandin E2 (PGE2), 20:5n-3, 22:5n-3, 22:6n-3, and n-3 PUFA. In vegetarians, plasma 20:4n-6 was significant positively related to TNF-α. LTB4 was significantly positively related to plasma 22:6n-3, and negatively associated with n-6 PUFA.

**Conclusion:**

Vegetarians have higher plasma n-6 PUFA and IL-6, but lower LTB4, n-3 PUFA, 22:6n-3, COX2 and PGE2 levels. It would seem appropriate for vegetarians to increase their dietary n-3 PUFA, while reduce dietary n-6 PUFA and thus reduce the risk of chronic inflammatory-related diseases.

## Introduction

It is widely recognized that overall mortality, ischemic heart disease mortality and overall cancer incidence are lower in vegetarians compared with omnivores [1, 2]. The dietary patterns of vegetarians as well as their healthful lifestyle practices are thought to at least partly explain these differences. Vegetarian diets are rich in fiber, magnesium, Fe^3+^, folic acid, vitamins C and E, n-6 polyunsaturated fatty acid (PUFA), phytochemicals, and antioxidants [3]. Another notable difference relates to the type and amount of fat in the diet. Vegetarian diets are slightly lower in total fat than omnivorous diets [4, 5]. Low intake of total fat, saturated fatty acids (SFA), and sodium and high intake of fiber, phytochemicals, and antioxidants in vegetarians is associated with low blood pressure and body mass index [3].

However, vegetarians diets are low in sodium, zinc, Fe^2+^, vitamins A, vitamins B_12_, and D, and n-3 polyunsaturated fatty acid (PUFA) [3, 6]. With respect to intake of essential fatty acid, especially n-3 PUFA, vegetarian diets appear to offer no advantages over omnivorous dietary patterns. It has been suggested that vegetarians could be at a significant disadvantage, as consumption of α-linolenic acid (18:3n-3) islow, resulting in limited conversion of 18:3n-3 to eicosapentaenoic acid (20:5n-3) and docosahexaenoic acid (22:6n-3) [7]. In addition, ovolacto vegetarian could get limited amount of 20:5n-3 and 22:6n-3, however, vegan diet almost has no 20:5n-3 and 22:6n-3, they may get tiny amount from algae and seaweed [4].

Eicosanoids, which are the mediators and regulators of inflammation, are generated from 20-carbon PUFA. Because inflammatory cells typically contain a high proportion of the n-6 PUFA arachidonic acid (20:4n-6) and low proportions of other 20-carbon PUFA [8], 20:4n-6 is a substrate of 2- and 4-series eicosanoids, which are associated with inflammation. Many anti-inflammatory pharmacotherapies are directed at inhibiting the production of these inflammatory mediators and thus possibilities exist for therapies that incorporate n-3 PUFA [9]. High level of n-3 PUFA in the diet can suppress the production of both tumor necrosis factor α (TNF-α) and interleukin 1β (IL-1β) [10]. Dietary supplementation with encapsulated fish oil rich in 20:5n-3 and 22:6n-3 has been shown to result in decreased synthesis of TNF-α and interleukin-1β (IL-1β) monocyte by in healthy subjects [10].

To date, no study reported the relationship of plasma fatty acids with blood inflammatory factors in Chinese vegetarians. Therefore, the purpose of the present study was to investigate the status of plasma fatty acids and to examine the potential relationship between fatty acids and plasma inflammatory factors in Chinese vegetarians and omnivores.

## Materials and methods

### Subjects

The study protocol was approved by the Ethics Committee, Zhejiang Hospital, Hangzhou, China, and all subjects were volunteers who gave their written consent prior to participation in the study. A total of 89 male vegetarians (aged 35.39 ± 12.2 year) were recruited in Linyin Temple, Hangzhou, China. A vegetarian was defined as someone who ate no red meat, fish and chicken, and had been following this diet for at least 6 months prior to the study. A total of 106 male omnivores (aged 36.62 ± 9.8 year) were recruited through a health check program during the period of October 2010 through March 2011 in the Zhejiang Hospital, Hangzhou, China. An omnivore was defined as someone who ate meat at least five times per week.

### Blood collection

Subjects attended the Zhejiang Hospital in the morning following an overnight fast. Subjects were allowed to sit relaxed for 10 min, the subject's weight, height, waist to hip ratio and blood pressure were measured. Then venous blood was taken in plain and EDTA vacuum tubes with 21-gauge needles. After blood collection, plasma samples were prepared quickly after blood was drawn, aliquoted into separate tubes and stored at -20°C until analysis.

### Laboratory measurements

Total lipid of plasma was extracted with chloroform:-methanol 1:1 (C:M, v/v) containing 10 mg/L of butylated hydroxytoluene (Tokyo Kasei Kogyo Co., Ltd. Japan). The plasma phospholipid (PL) fractions were separated by thin-layer chromatography. The methyl esters of the fatty acids of the plasma PL fractions were prepared using 0.9% H2SO4 in methanol containing 35 mg/L of n-Nonadecanoic acid (C19:0) (Chem Service, PA, USA) as internal standards. The fatty acid composition of plasma PL was determined by capillary gaseliquid chromatography using an Agilent 60 m_ 0.25 mm_ 0.25 mm column [11, 12]. IL-6, LTB4, COX2, PGE2 were determined by ELISA kits (R&D Systems, USA) through standard method in Clinical laboratory, Zhejiang Hospital, China.

### Statistical analysis

Data analyses were performed using SAS for Windows, version 9.1 (SAS Institute). All continuous variables were examined for normal distribution. Differences between the two groups for each outcome were analyzed using t-test. The associations between plasma fatty acid composition and inflammatory factors were determined by partial correlation, controlling for potential confounding factors. All data are expressed as mean ± SD. Differences between groups were considered to be statistically significant at *p* < 0.05.

## Results

Compared with omnivores, the weight was significantly lower in vegetarians (p < 0.001). IL-6 (p < 0.001) was significantly higher in vegetarians, while LTB4 (p < 0.001), COX2 (p < 0.001) and PGE2 (p < 0.001) were significantly lower in vegetarians (*P* = 0.005) (Table 1).

**Table 1:** **The demographic and biochemical measurements in vegetarians and omnivores**

	Vegetarians n = 89	Omnivores n = 106	P value
Age (year)	35.39 ± 12.2	36.62 ± 9.8	0.444
Height (m)	1.68 ± 0.06	1.72 ± 0.05	<0.001
Weight (kg)	66.96 ± 10.52	70.65 ± 9.62	0.012
BMI (kg/m^2^)	23.75 ± 3.27	23.76 ± 2.80	0.975
Log IL-1 (pg/mL)	0.11 ± 0.029	0.11 ± 0.044	0.430
Log IL-2 (pg/mL)	0.24 ± 0.11	0.27 ± 0.13	0.096
Log IL-6 (pg/mL)	2.33 ± 0.11	2.12 ± 0.14	<0.001
Log IL-10 (pg/mL)	1.01 ± 0.14	1.03 ± 0.21	0.503
Log TNF-α (pg/mL)	0.17 ± 0.058	0.16 ± 0.057	0.536
Log LTB4 (pg/mL)	1.84 ± 0.26	1.98 ± 0.20	<0.001
Log COX2 (mg/mL)	0.498 ± 0.147	0.610 ± 0.173	<0.001
Log PGE2 (pg/mL)	1.82 ± 0.150	2.08 ± 0.142	<0.001

Data was expressed as Mean ± SD.*P* < 0.05 indicate the significant difference between groups.

t test was used to test the differences between groups.

TNF-α: tumor necrosis factor-alpha, IL: interleukin, LTB4: Leukotriene B4, COX2: cyclo-oxygenase 2, PGE2: prostaglandin E2.

The plasma fatty acids composition was significantly different between vegetarians and omnivores. Compared with omnivores, the plasma 20:5n-3 (*p* < 0.001), 22:5n-3 (*p* < 0.001), 22:6n-3 (*p* < 0.001), and n-3 LC-PUFA (*p* < 0.001) were significantly lower in vegetarians, while, the plasma n-6 PUFA (*p* < 0.001), n-6/n-3 (*p* < 0.001), and 18:3n-3 (*p* < 0.001) were significantly higher in vegetarians (Table 2).

**Table 2:** **Plasma fatty acid composition in vegetarians and omnivores**

PL Fatty acids (% of total fatty acids)	Plasma ^a^		P value
	Vegetarians	Omnivores	
	n = 89	n = 106	
18:2n-6	25.94 ± 4.67	24.08 ± 3.12	0.001
18:3n-6	0.34 ± 0.20	0.10 ± 0.10	0.000
20:2n-6	1.33 ± 0.20	0.44 ± 0.24	0.000
20:3n-6	4.99 ± 2.51	2.46 ± 1.55	0.000
20:4n-6	15.04 ± 9.21	10.87 ± 2.73	0.000
22:2n-6	0.49 ± 0.45	0.22 ± 0.31	0.000
22:4n-6	0.64 ± 0.59	0.39 ± 0.27	0.000
18:3n-3	0.39 ± 0.29	0.19 ± 0.12	0.000
20:5n-3	0.83 ± 1.20	1.34 ± 1.13	0.003
22:5n-3	1.09 ± 0.42	1.23 ± 0.35	0.020
22:6n-3	2.12 ± 0.85	4.66 ± 1.45	0.000
n-6 PUFA	48.57 ± 7.02	38.84 ± 3.05	0.000
n-3 PUFA	4.45 ± 1.84	7.41 ± 2.07	0.000
n-6/n-3	12.33 ± 4.70	5.63 ± 1.68	0.000

Data was expressed as Mean ± SD.

^a^All fatty acids are significantly different between two groups (*p* < 0.05).

The difference between the vegetarians and omnivores is determined by using general linear model controlled potential confounding factors (Age and BMI).

PUFA: polyunsaturated fatty acids, MUFA, monounsaturated fatty acids.

O: omnivores, V: vegetarians, PL: phospholipids.

We used the partial correlations to test the associations between inflammatory factors and plasma fatty acids. In vegetarians, plasma 20:4n-6 (r = 0.241, *p* < 0.05) was significantly positively correlated with TNF-α. LTB4 was significantly positively correlated with plasma 22:6n-3 (r = 0.241, *p* < 0.05), and negatively correlated with n-6 PUFA (r = -0.270, *p* < 0.05) (Table 3).

**Table 3:** **Partial correlations between plasma fatty acid compositions and plasma inflammatory factors**

		20:4n-6	20:5n-3	22:6n-3	n-3 PUFA	n-6 PUFA	n-6/n-3
IL-1	V	0.125	-0.17	-0.1	-0.19	0.102	-0.19
	O	0.065	0.051	0.045	0.086	-0.112	0.11
IL-2	V	0.098	0.127	-0.13	-0.06	0.039	-0.06
	O	-0.063	-0.093	0.016	-0.04	0.007	-0.039
IL-6	V	-0.02	-0.02	-0.12	-0.13	-0.03	-0.1
	O	0.079	0.136	0.053	0.124	-0.15	0.155
IL-10	V	-0.14	0.037	0.041	0.083	-0.2	0.132
	O	-0.112	-0.154	-0.047	-0.171	-0.042	-0.15
TNF-α	V	0.241*	-0.09	-0.13	-0.14	0.207	-0.19
	O	0.071	-0.008	0.061	0.059	-0.096	0.08
LTB4	V	-0.08	-0.03	0.316*	0.077	-0.27*	0.136
	O	0.005	0.08	-0.122	-0.096	-0.14	-0.056
PGE2	V	0.106	-0.07	0.054	0.086	-0.04	0.08
	O	0.163	0.064	0.049	0.063	0.097	0.035
COX2	V	-0.11	-0.07	0.081	0.024	-0.17	0.079
	O	-0.045	0.123	0.113	0.116	-0.201	0.156

The associations of plasma PL fatty acids compositions with plasma inflammatory factors centration was tested by using pearson partial correlation model after controlling confounding factor(age, BMI).

Coef, partial correlation coefficient, O: omnivores, V: vegetarians, PL: phospholipids.

*p < 0.05.

## Discussions

In the present study, we found that vegetarians have higher plasma n-6 PUFA, n-6/n-3, 18:3n-3 and IL-6, lower plasma 20:5n-3, 22:5n-3, 22:6n-3, n-3 PUFA, LTB4, COX2 and PGE2.In vegetarians, plasma 20:4n-6 was significantly positively correlated with TNF-α. LTB4 was significantly positively related with plasma 22:6n-3.

Vegetarian diets are slightly lower in total fat than omnivorous diets [4]. Vegetarian diets are rich in n-6 PUFA, but, low in n-3 PUFA [3]. Ovolacto vegetarians consume minimal amounts of 20:5n-3 and varying amounts of 22:6n-3 from eggs, milk, and dairy products. Vegans consume negligible amounts of n-3 PUFA and rely entirely on in vivo biosynthesis of n-3 PUFA from the precursor 18:3n-3, but conversion via desaturation and elongation, especially to 22:6n-3, is not efficient [12]. Previous study has shown conversion of 18:3n-3 to 20:5n-3 varying from 6-21% to much lower values(0.1-0.2%), or undetectable 22:6n-3 synthesis [13]. In the present study, we found that vegetarians have higher plasma PL n-6 PUFA, n-6/n-3, and 18:3n-3, while lower plasma PL 20:5n-3, 22:5n-3, 22:6n-3, and n-3 long-chain (LC)-PUFA. Lack of 20:5n-3 and 22:6n-3 in vegetarian diets is reflected in reduced amounts of these fatty acids in platelets, RBC, and plasma [14]. Thus, uptake of preformed 22:6n-3 from the diet may be critical for maintaining adequate membrane 22:6n-3 concentrations in vegetarians [14]. However, we did not collect socio-economic information for all subjects. Further studies are required to examine whether socio-economic information may modify the observed associations. In addition, our results showed a high composition of LC-PUFA compared to previous report. The methods used for fatty acids determination and population discrepancy may help explain this inconsistent result.

Previous studies and our present study demonstrated that n-3 PUFA and 22:6n-3 in plasma or red blood cell was significantly lower in vegetarians. Sanders et al. reported that the proportions of 22:6n-3 in plasma, blood cells, breast milk, and tissues are substantially lower in vegans and vegetarians compared with omnivores [15]. Kornsteiner et al. also demonstrates that vegetarians and vegans, who do not eat meat or fish, tend to have very low or negligible intakes of 20:5n-3 as well as 22:6n-3 [14]. Fokkema et al. investigated the PUFA status of Dutch vegans and omnivores in erythrocyte membranes. They found that 20:5n-3 and 22:6n-3 was significantly reduced in vegans compared with omnivores, while, a higher 22:5n-3 content compared with the Dutch omnivores [16], which is consistent with the present results. A study investigating plasma n-3 PUFA of British meat-eating, vegetarian, and vegan men showed that 20:5n-3, and 22:6n-3 were markedly decreased [17]. Li et al. demonstrated a decreased content of 20:5n-3, 22:6n-3 and total n-3 PUFA in the Australian vegetarian group [18]. Sanders et al. showed that the erythrocytes from vegans contained lower proportions of 20:5n-3, 22:5n-3 and 22:6n-3 and higher proportions of 18:2n-6, 20:2n-6 and 22:4n-6 [19]. Therefore, the low content of plasma PL n-3 PUFA reflects the limited n-3 PUFA dietary intake in vegetarians. The unbalanced n-6:n-3 ratio and the limited dietary sources of 20:5n-3 and 22:6n-3 in vegans and vegetarians led to reductions in 20:5n-3, 22:5n-3, 22:6n-3 and n-3 PUFA in PC, PS and PE compared with omnivores and semi-omnivores [14]. Vegetarian people are suggested to supplement fish oil or other food rich in n-3 PUFA to increase the level of n-3 PUFA in tissues [14, 20].

Inflammation plays a central role in development of atherosclerosis, CVD and type 2 diabetes [21]. In the present study, we found that vegetarians have higher IL-6 concentrations. The possible mechanisms behind these associations are not fully understood. It was showed that elevated concentrations of pro-inflammatory cytokines such as IL-6 and acute phase reactants such as C-reactive protein are in turn independent risk factors for the development of type 2 diabetes [21] and CVD [22]. It has been shown that IL-6 may induce antagonists to inflammatory cytokines and therefore play a role in terminating inflammation [23]. The soluble TNF receptors, which are derived by proteolytic cleavage from TNF cell-surface receptors after induction by cytokines such as TNF, IL-6, IL-1β, or IL-2 [24], bind to TNF and attenuate its bioactivity. Furthermore, we found LTB4, COX2 and PGE2 were lower in vegetarians. Interestingly, cell culture demonstrated that LTB4 synthesis can be modulated by the fatty acid composition of membrane phospholipids, which can be altered by dietary fatty acids [25]. Previous study reported that dietary supplements rich in n-3 PUFA reduce the concentrations of PGE2 and increase the synthesis of PGE3, which are believed to be less inflammatory. PGE2 and PGE3 both induce COX-2 mRNA via similar signaling mechanisms [26]. It was shown that increasing the n-3 content of membrane phospholipid results in a decrease in mitogen-induced PGE2 synthesis.

This study demonstrated that replacement of n-6 PUFA with n-3 PUFA in cell membranes can result in a decreased cellular response to mitogenic and inflammatory stimuli [26].

Dietary factors substantially modulate inflammation and cardiovascular risk [27]. Long-chain n-3 PUFA reduces cardiovascular events and exerts well-established anti-inflammatory effects [28]. While, an increased ratio of dietary n-6 to n-3 PUFAs has been linked to the risk of chronic inflammatory diseases [28]. In the present study, we observed a light association between 22:6n-3 and IL-6. Interestingly, plasma 20:4n-6 was significantly positively correlated with TNF-α in Chinese male vegetarians. In consistent with our results, a cross-sectional study of dietary n-3 and n-6 fatty acids and inflammatory markers also observed statistically significant inverse associations between n-3 fatty acid intake and plasma levels of soluble TNF receptors 1 and 2. The associations were restricted to the LC PUFA 20:5n-3 and 22:6n-3 and not 18:3n-3 [29]. At low levels of n-3 PUFA intake, n-6 PUFA are associated with high levels of inflammatory markers, yet at high levels of n-3 fatty acid intake, the combination of both types of fatty acids is related to the lowest levels of inflammation [29]. Despite a lower dietary PUFA intake, obese individuals have higher proportions of 20:4n-6 in their adipose tissue than do their non obese twins, which render their adipocytes more vulnerable to inflammation [30]. Human trials reported that n-3 fatty acid intake decreased inflammation. LC n-3 PUFA intakes were inversely associated with plasma concentrations of IL-6 and matrix metalloproteinase-3. Non-fried fish consumption was found inversely related to C-reactive protein and IL-6; and fried fish was observed being inversely related to soluble intercellular adhesion molecules-1 (sICAM-1) but not associated with other biomarkers [31].

Intervention study showed that N-3 PUFA supplementation reduced plasma concentrations of soluble intercellular adhesion molecule-1, but had no significant effects on soluble vascular cell adhesion molecule-1, soluble P-selectin, or soluble E-selectin [32]. Treatment with LC n-3 PUFA favorably modulated adipose tissue and systemic inflammation in severely obese non-diabetic patients and improved lipid metabolism [27]. Perioperative fish oil infusions significantly increased PUFA concentrations in platelet and atrial tissue membranes within 12 h of the first FO administration and decreased biological and clinical signs of inflammation [33]. However, data from experimental studies of dietary n-3 PUFA and inflammatory markers reported quite conflicting results [34]. These experiments may be influenced by cell purification and culturing procedures and depend on cell origin and cell type, as TNF-α and IL-6 are produced by a variety of cells.

One possible biological mechanism underlying the beneficial effects of n-3 PUFA on inflammation and endothelial function is that these fatty acids compete with n-6 fatty acids for prostaglandin and leukotriene synthesis at the cyclooxygenase and lipoxygenase level [31]. n-3 PUFA from fish or fish oil modulate prostaglandin metabolism by increasing prostaglandin E_3_, thromboxane A_3_ and leukotriene B_5_ and by decreasing production of thromboxane A_2_ and leukotriene B_4_ formation [35]. Another suggested mechanism is that n-3 PUFA may react with active oxygen species because of their multiple double bonds and lead to a decreased production of hydrogen peroxide. Hydrogen peroxide is a critical activator of the nuclear factor-κB system of transcription factors that controls the coordinated expression of adhesion molecules and of leukocyte-specific chemoattractants upon cytokine stimulation [36]. Furthermore, the activities of the Δ6 and Δ5-desaturase and the activity of the cyclooxygenase are inhibited by both n-3 and n-6 PUFA [37]. Through this mechanism, high intake of PUFA could reduce inflammatory mediators through modulating inflammatory gene expression in immune cells [38]. PUFA may also modulate cytokine production or the release of the soluble TNF receptors through eicosanoid-independent pathways, for example, by influencing membrane composition and fluidity, affecting signal transduction processes or second messenger molecules, or binding to or affecting nuclear receptors such as the peroxisome proliferator receptors or nuclear factor-kB [29, 39].

In conclusion, vegetarians have higher IL-6 and n-6 PUFA, but lower n-3 LC-PUFA, 22:6n-3, LTB4, COX2 and PGE2 levels. It would seem appropriate for vegetarians to increase their dietary 22:6n-3, while reduce dietary n-6 PUFA and thus reduce the risk of chronic inflammatory-related diseases.
